# Development and validation of a methotrexate adherence assay

**DOI:** 10.1136/annrheumdis-2019-215446

**Published:** 2019-06-05

**Authors:** James Bluett, Isabel Riba-Garcia, Suzanne M M Verstappen, Thierry Wendling, Kayode Ogungbenro, Richard D Unwin, Anne Barton

**Affiliations:** 1 Versus Arthritis Centre for Genetics and Genomics, Centre for Musculoskeletal Research, The University of Manchester, Manchester, UK; 2 NIHR Manchester Biomedical Research Centre, Manchester University Hospitals NHS Foundation Trust, Manchester Academic Health Science Centre, Manchester, UK; 3 Centre for Advanced Discovery and Experimental Therapeutics (CADET), Division of Cardiovascular Sciences, The University of Manchester, Manchester, UK; 4 Versus Arthritis Centre for Epidemiology, Centre for Musculoskeletal Research, The University of Manchester, Manchester, UK; 5 Centre for Applied Pharmacokinetic Research, Division of Pharmacy and Optometry, University of Manchester, Manchester Academic Health Science Centre, Manchester, UK

**Keywords:** methotrexate, assay, adherence, rheumatoid arthritis, pharmacokinetic

## Abstract

**Background:**

The first-line therapy for rheumatoid arthritis (RA) is weekly oral methotrexate (MTX) at low dosages (7.5–25 mg/week). However, ~40% of patients are non-adherent which may explain why some do not respond and need to start more expensive biological therapies. To monitor adherence more accurately and develop strategies to improve it, a validated objective MTX adherence test is required.

**Objective:**

To develop and validate the diagnostic accuracy of a novel MTX adherence assay using high-performance liquid chromatography–selected reaction monitoring– mass spectrometry (HPLC-SRM-MS) based biochemical analysis of drug levels.

**Methods:**

20 patients with RA underwent MTX pharmacokinetic assessment using HPLC-SRM-MS MTX plasma concentration analysis over a 6-day period. Directly observed therapy was the reference standard. Pharmacokinetic model validation was performed using independent plasma samples from real-world patients (n=50) with self-reported times of drug administration. Following assay optimisation, the sensitivity of the assay to detect adherence was established using samples from an observational cohort study (n=138).

**Results:**

A two-compartment pharmacokinetic model was developed and validated. Simulations described the sensitivity required for MTX detection over 7 days; subsequent assay optimisation and retesting of samples confirmed that all patients were correctly identified as MTX adherers. Using real-world samples, the assays sensitivity was 95%.

**Conclusion:**

Non-adherence to MTX is common and can have a significant effect on disease activity. HPLC-SRM-MS plasma analysis accurately detects MTX adherence. The validated objective test could easily be used in clinic to identify patients requiring adherence support.

**Key messages**

**What is already known about this subject?**

- About 40% patients are non-adherent to methotrexate, up until now there has been no objective direct method to measure methotrexate non-adherence.

**What does this study add?**

- This study demonstrated that methotrexate adherence can be measured using a novel high-performance liquid chromatography–selected reaction monitoring–mass spectrometry assay which could easily be used in the clinic.

**How might this impact on clinical practice or future developments?**

- In the future, the developed test could be used as part of a biofeedback tool to improve non-adherence healthy behaviour in patients.

## Introduction

The antifolate drug methotrexate (MTX) is the first-line therapy for rheumatoid arthritis (RA) in European and American guidelines.^1 2^ MTX response is not, however, universal; response is likely to be influenced by a number of factors including clinical, psychological and biological features. MTX adherence has been shown to be a modifiable health behaviour that affects RA response.^3–5^ A recent systematic review identified adherence rates varying between 59% and 107%, with the latter figure representing MTX overdosage, a form of non-adherence.^6^ This wide range of adherence rates is likely, in part, to be due to study heterogeneity, with a number of different adherence definitions applied and the use of imprecise indirect measures of adherence in these studies, such as questionnaires, which are more subjective. Failure of MTX and a further conventional synthetic disease-modifying antirheumatic drug to control disease makes patients eligible to receiving more expensive biological therapy, but it has not previously been possible to determine if a patient is non-responsive to MTX as a consequence of non-adherence. There is, therefore, a need to measure adherence directly to facilitate more precise and objective measurements, to add to the clinicians arsenal to detect non-adherence and help to open up honest discussions and supportive interventions with patients. Such a direct method of measurement is likely to involve the detection of MTX itself or an MTX metabolite. This approach has been successfully developed for the objective detection of hydroxychloroquine non-adherence.^7^

In oncology, MTX is used at high doses in leukaemia (eg, 1000 mg/m^2^) where it is routine practice to measure MTX levels.^8^ The commonly used immunoassays cross-react with other substances, reducing their specificity and lack sensitivity for the measurement of MTX in patients receiving low-dose, weekly regimens such as those used to treat RA.^9^ The use of high-performance liquid chromatography–selected reaction monitoring–mass spectrometry (HPLC-SRM-MS) for the detection of adherence has the particular advantage of a high sensitivity that is required for drugs, such as MTX, that are administered in low dosages. Importantly, direct testing of drug levels using HPLC-SRM-MS has been shown to improve medication adherence in other diseases such as hypertension, resulting in improved treatment response.^10^

The aim of this study was to develop and investigate the diagnostic accuracy of an HPLC-SRM-MS assay for the detection of MTX drug levels as a direct measurement of adherence. This required (1) the development of an MTX adherence assay, (2) a pharmacokinetic (PK) study of MTX to evaluate the ability of the assay to measure adherence, (3) validation of the PK model in an observational cohort, (4) further optimisation of the assay to the required performance criteria and finally (5) investigation of the ability of the assay to detect adherence in samples from real-world patients.

## Methods

### Test methods

**Figure 1:**
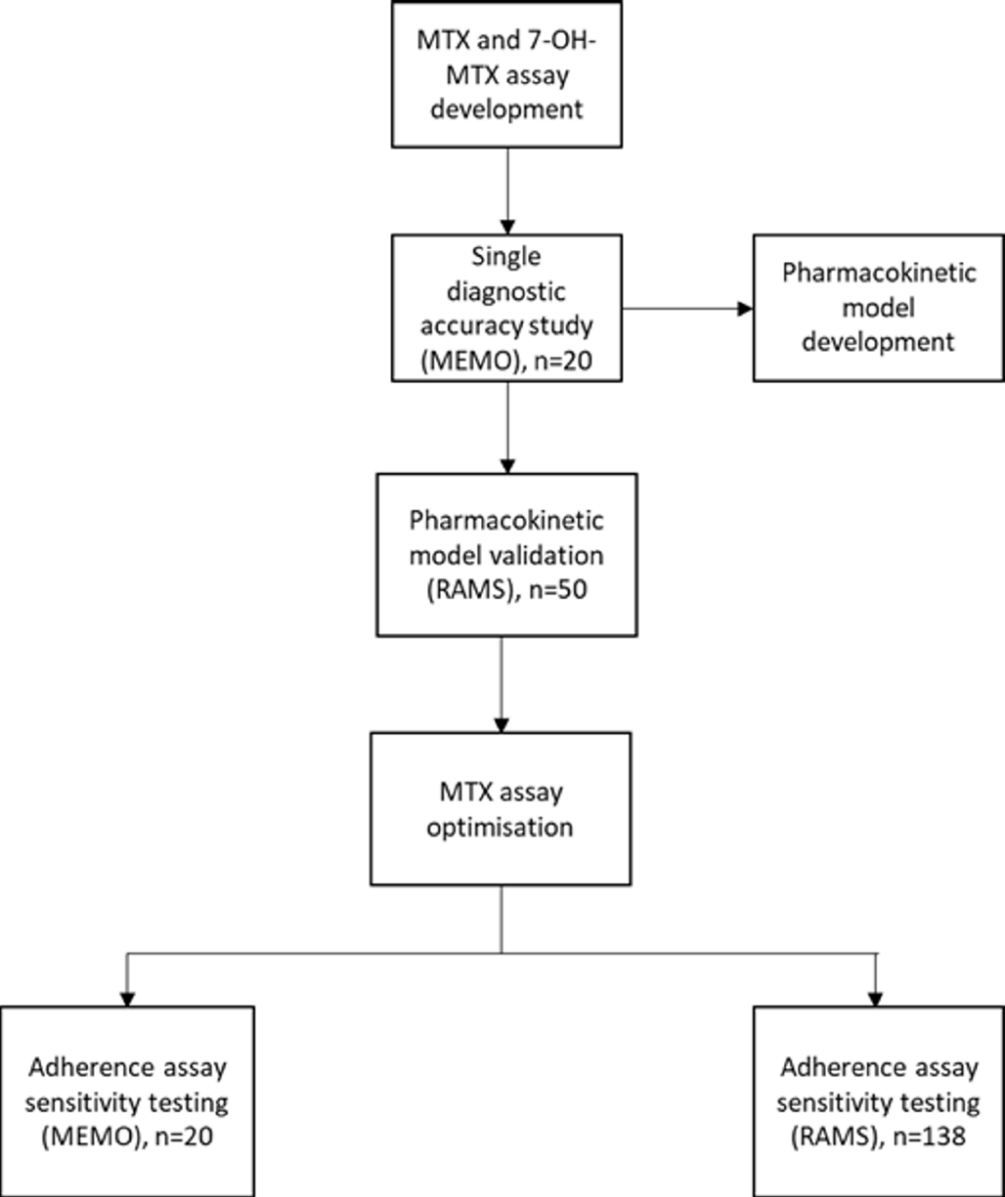
Overview of study schedule. 7-OH-MTX, 7-hydroxy-MTX; MEMO, Measurement of MTX and 7-OH-MTX metabolites in urine of patients with rheumatoid arthritis; MTX, methotrexate; RAMS, Rheumatoid Arthritis Medication Study.

An overview of the study procedures is shown in figure 1. Prior to data collection the index test, an HPLC-SRM-MS assay for the detection of MTX and its major metabolite 7-hydroxy-MTX (7-OH-MTX), was developed using known concentrations of MTX and 7-OH-MTX spiked into plasma samples and subsequently optimised (for sample preparation, chromatographic and MS conditions, see online supplementary S1).

10.1136/annrheumdis-2019-215446.supp1 Supplementary data

The assay had an initial lower limit of quantification (LLOQ) of 0.5 nM and 0.75 nM for MTX and 7-OH-MTX, respectively. Samples were tested in triplicate and the measurement accepted if coefficient of variation (CV) ≤15% and the result was within the calibration curve. Samples not passing quality control were excluded from the analysis. Mean measurements were multiplied by their dilution factor to obtain the final concentration. The assay was subsequently validated for measurement in serum samples; drug-free serum was prepared as per the plasma preparation protocol. The linearity, LLOQ, carryover and precision of the assay were determined in the plasma samples.

### Patient and public involvement

The study design was developed in collaboration with a Research User Group (RUG) of patients and carers living with musculoskeletal conditions, including RA. The RUG assessed the patient information resources that were appropriately worded and assessed the burden of the study to the participants.

### Study design and participants

The single-centre diagnostic accuracy study (Measurement of MTX and 7-OH-MTX metabolites in urine of patients with rheumatoid arthritis; the MEMO study) included patients who were at least 18 years of age, had a physician diagnosis of RA and were commencing MTX as part of their usual care. Patients were identified from the Rheumatoid Arthritis Medication Study (RAMS) study, a 1-year prospective multicentre observational study in the UK designed to identify predictors of response to MTX in patients with RA.^11^ Eligible and consenting patients recruited to RAMS between 2014 and 2015 were invited to participate in the MEMO study. Patients were excluded if they had a contraindication to MTX as per the Summaries of Product Characteristics.^12^ Patients were invited sequentially according to the proximity of the hospital providing their care to the National Institute for Health Research (NIHR) Clinical Research Facility (CRF) in Manchester, UK, to reduce the travel burden for potential study participants. Clinical and demographic data recorded included age, gender, weight, Disease Activity Score-28, medication and dosage. Blood samples were taken to measure creatinine, calculated estimated glomerular filtration rate (calculated using Modification of Diet in Renal Disease) and albumin. A recruitment size of 20 participants was deemed to be sufficient based on previous studies developing similar drug level adherence assays.^13 14^

### MEMO study schedule and sample collection

Participants were screened and attended three visits at the CRF, Manchester, UK. Plasma samples were collected for the measurement of MTX concentrations from all patients prior to and following directly observed therapy of MTX at baseline (the reference standard). Samples were collected in K_2_EDTA collection tubes at 0, 1, 2, 4, 8, 16, 24 hours and on 2 subsequent days within 7 days of observed MTX ingestion at a date/time convenient to the participant. Samples were placed on ice for a maximum of 30 min prior to sample preparation. The plasma fraction was prepared immediately by centrifugation at 1500g for 10 min at 4°C. Samples were divided into aliquots (0.5 mL) in cryovials (Greiner) and frozen by placing in a −80°C freezer. Samples were labelled with the patient ID, date and time of collection. Clinical information and reference standard results were available to the performers/readers of the index test.

### PK model and adherence test validation in real-world samples

PK model validation was undertaken, to replicate the model, using independent samples from the RAMS study. Briefly, a specially designed diary was used to collect adherence data. Each week for 26 weeks, patients recorded their MTX use including day and time of ingestion and/or usual time of MTX ingestion in the case report form. Three-month and 6-month blood samples were collected, the date/time of sampling recorded and the samples were posted to the coordinating centre in Manchester. Samples were processed as described previously and stored at −80°C until measurement. Three-month and 6-month samples were used to validate the PK model and ability of the assay to measure adherence, respectively.

### Analysis

#### Development and validation of an MTX PK model

A population PK model was developed using mixed-effect modelling software NONMEM version 7.3.0 (ICON Development Solutions, Hanover, Maryland, USA).^15^ Estimation of the population median and variance parameters was performed using a Bayesian approach and uninformative priors for all parameters. Based on visual inspection of the concentration–time profiles of MTX and 7-OH-MTX and previously published data, a two-compartment model for MTX and one-compartment model for 7-OH-MTX was fitted to the data. For the metabolite 7-OH-MTX, apparent formation and clearance of 7-OH-MTX were estimated as previously described.^16^ Covariates (body weight and serum creatinine levels) for the model parameters were tested to determine whether any part of the variability in the parameters was explained. PK parameters were reported with their relative SE to provide an estimate of uncertainty in the parameters. The PK model was validated by plotting, over time, the dose-normalised observed concentrations of the sparse RAMS samples along with the median of the predicted concentrations and a 90% prediction interval. Simulations were performed to predict the proportion of patients with detectable concentrations of both MTX and 7-OH-MTX over time to inform required assay sensitivity to detect adherence for a given dose and whether MTX or 7-OH-MTX is the most sensitive analyte in plasma samples.

#### Assay sensitivity analysis

Following PK model validation, simulations were used to determine adherence cut-offs required for the correct detection of adherence according to dose of MTX ingested with a proportion of samples predicted to be true positives ≥80%. Assay optimisation was undertaken to improve the LLOQ (online supplementary S2).

Six-month RAMS blood samples were used to assess the sensitivity of the assay to detect adherence. Samples were measured in triplicate and rejected if CV ≥25%; MTX-d_3_ was not detected in two or more samples or the measurement was outside the calibration range when taking into account the expected minimum concentration according to the PK model and the patients’ MTX dose. Triplicates where one sample failed were included if the sample failed due to non-detection of the internal standard MTX-d_3_. Mean measured concentration was used to detect adherence.

## Results

### Clinical characteristics

Twenty RA patients were recruited (see online supplementary S3 for the CONSORT (Consolidated Standards of Reporting Trials) 2010 flow diagram). The baseline characteristics of the MEMO cohort is shown in table 1.

**Table 1:** Baseline demographic and clinical details for the MEMO cohort

Baseline characteristic	Median (IQR)
Age (years)	65.5 (54–70)
Female gender (%)	65
Weight (kg)	76.9 (67.3–85.4)
Serum creatinine (µM)	71.5 (67.0–79.0)
eGFR (mL/min/1.73 m^2^)^*^	84 (76–99)
Serum albumin (g/L)	37 (37–39)
MTX dose (mg/week)	15 (7.5–25)^†^
Taking concomitant folic acid (%)	100
Taking concomitant NSAIDs (%)	20

*Calculated using the MDRD eGFR calculation.

†Median (range).

MDRD, Modification of Diet in Renal Disease; MEMO, Measurement of MTX and 7-OH-MTX metabolites in urine of patients with rheumatoid arthritis; MTX, methotrexate; NSAID, non-steroidal anti-inflammatory drug; 7-OH-MTX, 7-hydroxy-MTX; eGFR, estimated glomerular filtration rate.

### PK profile

**Figure 2:**
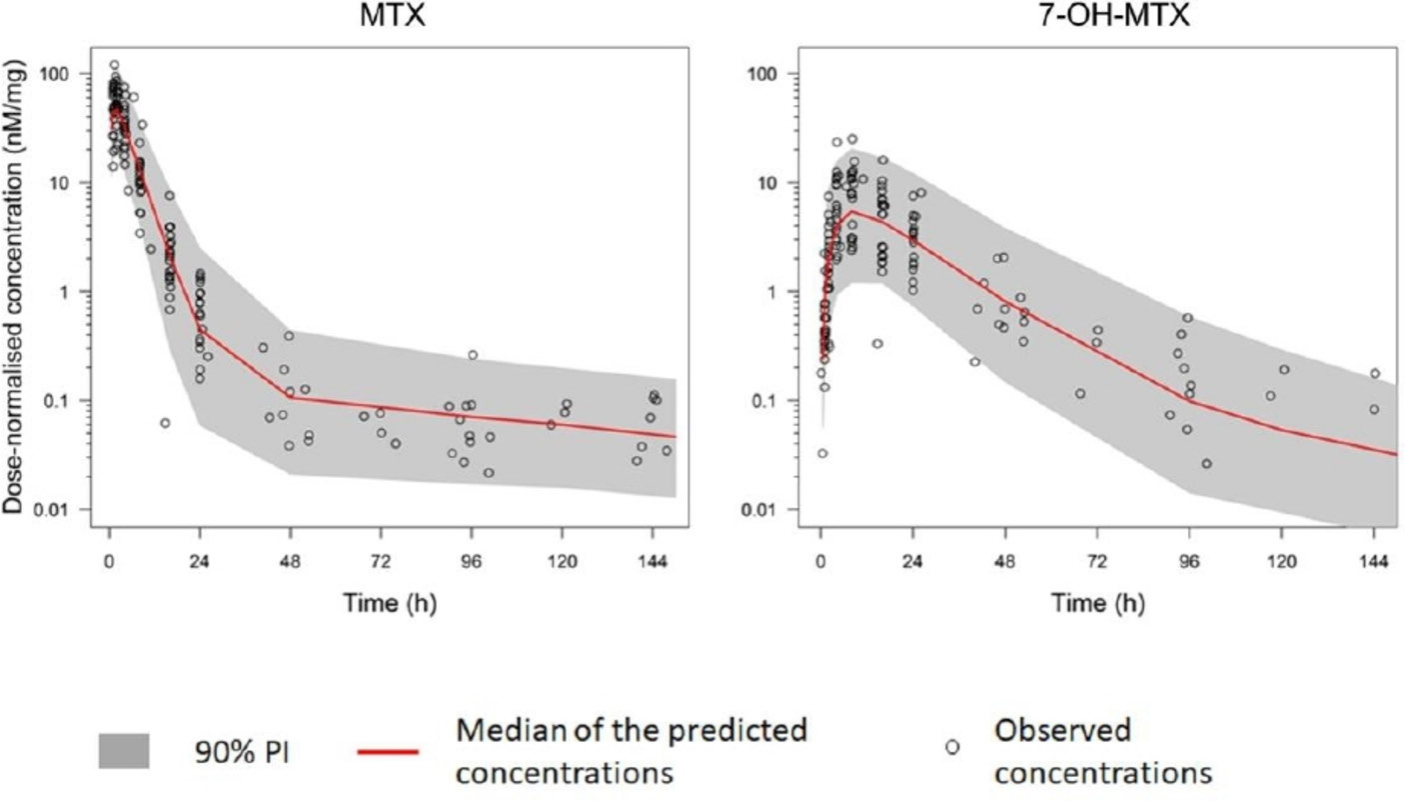
Visual predictive check for MTX and 7-OH-MTX. Observed concentrations are log-transformed dose-normalised (nM/mg) for MTX and 7-OH-MTX. 7-OH-MTX, 7-hydroxy-MTX; MTX, methotrexate; PI, prediction interval.

In total, 174 plasma samples (range 7–9 per patient) were collected from the MEMO study and measured in triplicate from 20 patients with blank plasma samples in each assay run. The median time from MTX ingestion to last plasma sample was 101 hours (IQR: 94–142 hours). No MTX-free plasma samples falsely detected MTX. Rejection of samples due to high CV or measurement less than LLOQ was 1.1 and 9.8 for MTX and 2.3% and 12.6% for 7-OH-MTX, respectively. MTX absorption was rapid, with plasma concentrations peaking at around 2 hours after oral administration. A two- compartment model for MTX and one-compartment model for 7-OH-MTX was fitted to the data (online supplementary S4). The effect of serum creatinine levels on the systemic clearance of MTX was negligible and was therefore not included in the model. The PK parameters for MTX and 7-OH-MTX are available in online supplementary S5. Intersubject variability was highest for the apparent fraction of MTX converted to 7-OH-MTX. The visual predictive check demonstrated that the model captured adequately the observed data for MTX and 7-OH-MTX as shown in figure 2.

**Figure 3:**
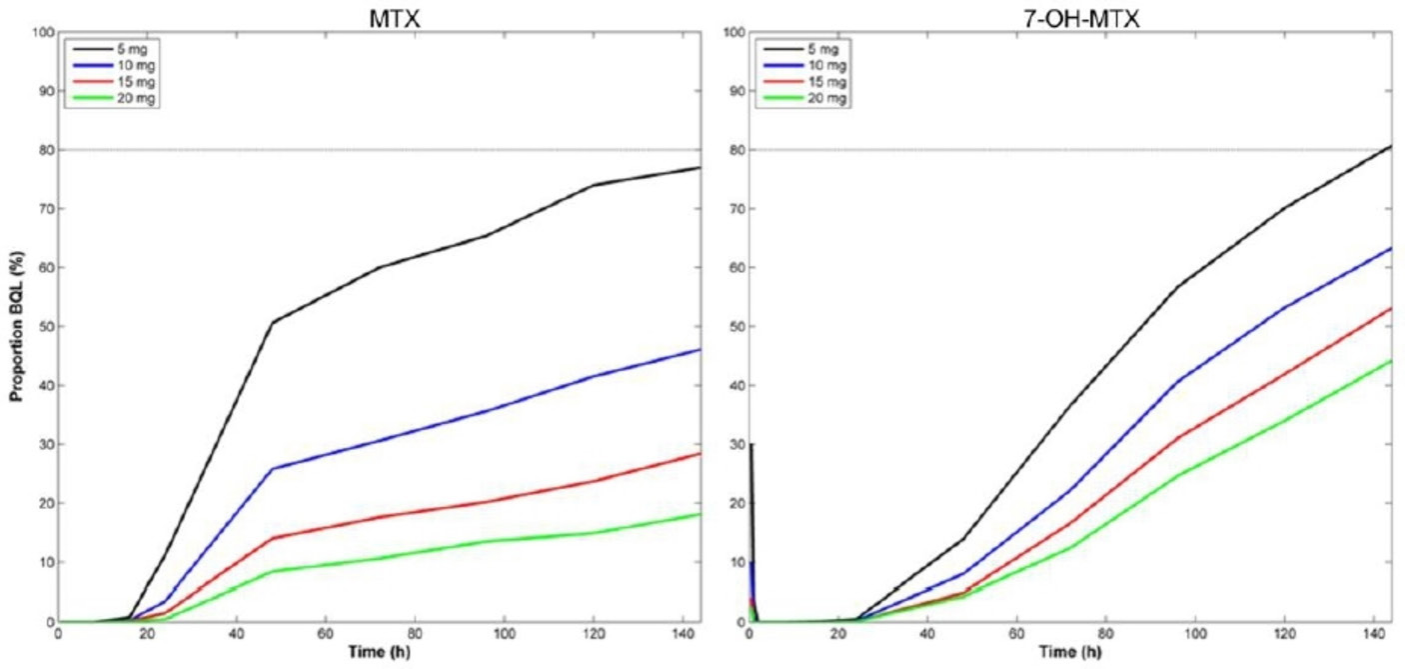
Simulated data of 1000 hypothetical individuals showing the proportion of subjects with predicted concentrations of MTX/7-OH-MTX below the LLOQ (BLQ) for 5, 10, 15 and 20 mg MTX. 7-OH-MTX, 7-hydroxy-MTX; BLQ, below the lower limit of quantification; LLOQ, lower limit of quantification; MTX, methotrexate.

Simulation data of 1000 hypothetical individuals after ingesting 5, 10, 15 and 20 mg MTX to predict the proportion of subjects with measured MTX and 7-OH-MTX levels below the LLOQ is shown in figure 3. The results demonstrate that while at 144 hours (6 days) following ingestion of 15 mg MTX, 72% of adherent patients are predicted to have measurable MTX, only 70% of adherent patients are predicted to have measurable MTX levels at 72 hours after ingestion of a lower MTX dose (ie, 10 mg), limiting the interpretation of the assay at lower doses. Further optimisation to improve the lower level of quantification was therefore undertaken. MTX was found to be a more accurate surrogate marker of adherence compared with 7-OH-MTX with a lower proportion of subjects that are predicted to be below the LLOQ for all dose ranges of MTX. Early after ingestion of MTX, 75% of subjects have undetectable 7-OH-MTX, due to the delay in hepatic metabolism of MTX to 7-OH-MTX.

### PK model validation

**Figure 4:**
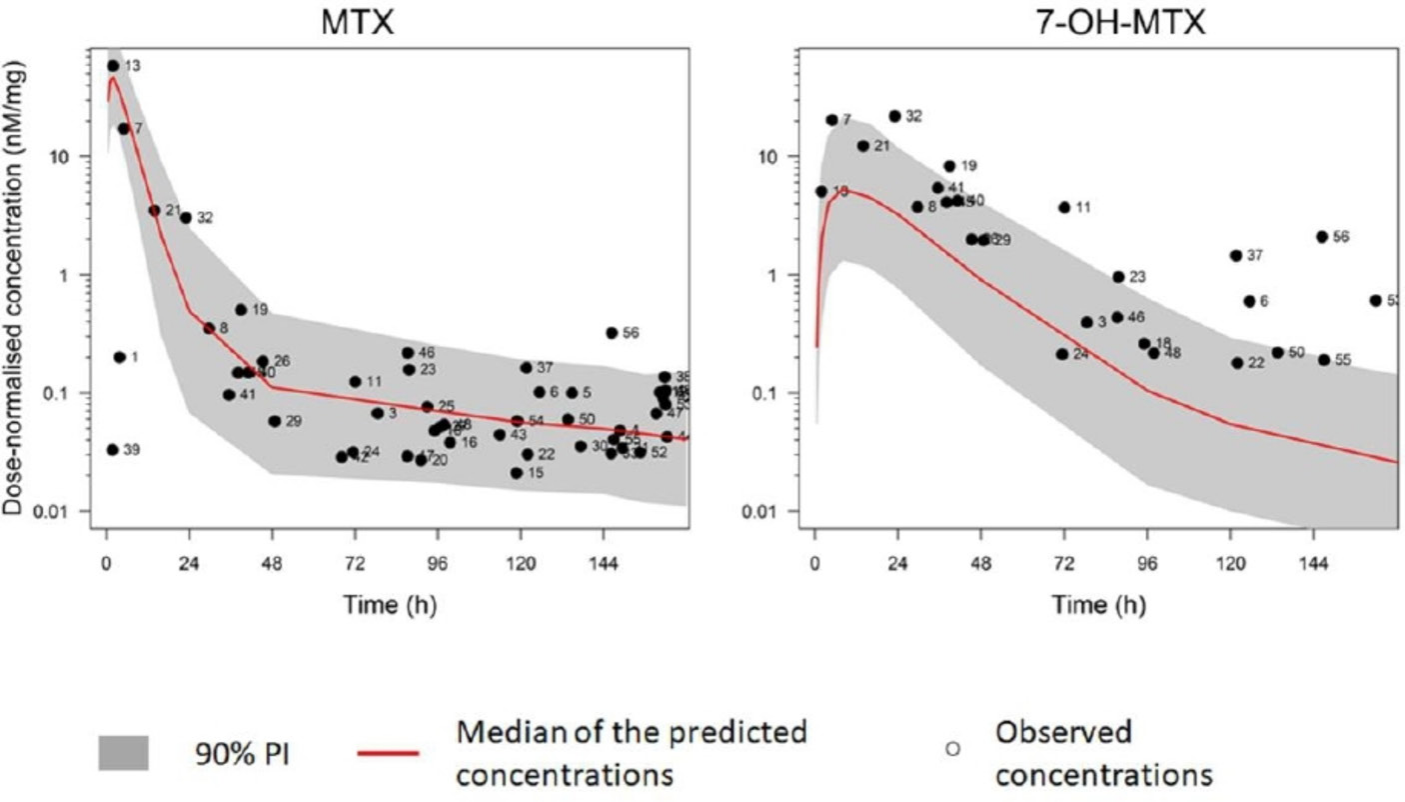
Log-transformed dose-normalised median and 90% PI MTX and 7-OH-MTX concentration developed from the MEMO study overlaid with individual dose-normalised MTX and 7-OH-MTX concentrations observed from the RAMS study. 7-OH-MTX, 7-hydroxy-MTX; MEMO, Measurement of MTX and 7-OH-MTX metabolites in urine of patients with rheumatoid arthritis; MTX, methotrexate; PI, prediction interval; RAMS, Rheumatoid Arthritis Medication Study.

In total, 51 plasma samples were collected where time of MTX ingestion was diarised and date/time of venepuncture was recorded from the RAMS cohort (median 99.6 hours; IQR: 58.5–147.6). Baseline clinical and demographic characteristics are shown in table 2. Of 51 samples, two showed undetectable levels of MTX (4%); of these, one sample was taken 58 days after the patient had stopped MTX but they had continued to participate in RAMS. Review of the diary for this patient revealed that the individual stopped MTX as the patient was unaware to continue treatment; following venepuncture, the patient restarted MTX and noted this in the diary; therefore, the patient was included in the study as having had taken MTX on the day of venepuncture. The sample was subsequently removed from analysis. The other sample was taken 148 hours after 20 mg MTX ingestion, a time later than the last time used for simulation. In comparison to MTX, 7-OH-MTX was undetectable in 26 (51%) of plasma samples. Figure 4 shows the median predicted dose-normalised concentration of MTX/7-OH-MTX with 90% prediction interval over time developed from the MEMO study with individual dose-normalised concentrations measured from the RAMS samples.

**Table 2:** Baseline clinical and demographic characteristics of the RAMS cohort

Baseline characteristic	Median (IQR)	Missing (n)
Age at venepuncture date (years)	62 (56–72)	0
Days between MTX commencement and venepuncture date	92 (88–105)	
Female gender (%)	55	0
Weight (kg)	76.9 (61.2–83.8)	3
Serum creatinine (µM)	67.5 (60.0–79.0)	5
Baseline DAS-28	4.61 (3.83–5.66)	2
MTX dose (mg/week)	20 (10–25)^*^	0

*Median (range).

DAS-28, Disease Activity Score-28; MTX, methotrexate; RAMS, Rheumatoid Arthritis Medication Study.

### Assay sensitivity analysis

Simulations were performed and confirmed that an LLOQ of 0.1 nM was sufficient to detect adherence at 7 days for each dose of MTX ≥5 mg with a predicted proportion above the LLOQ of ≥80% (online supplementary S6a-g). Based on these results, the adherence cut-offs were as shown in table 3.

**Table 3:** Oral MTX dose (mg/week) and MTX adherence limit (nm) with >80% proportion of subjects who are adherent according to the 1000 hypothetical subjects ingesting MTX 168 hours prior to blood sampling

**MTX dose (mg/week)**	**Adherence limit (nM)**
5	0.1
7.5	0.15
10	0.2
12.5	0.25
15	0.25
17.5	0.25
20	0.25
22.5	0.5
25	0.5

MTX, methotrexate.

The assay was subsequently reoptimised for sensitivity to generate a new LLOQ of 0.1 nM by further optimising the mass spectrometer parameters (online supplementary S2). Seventeen previously false-negative MEMO samples that were rejected due to measuring below the LLOQ were retested using this optimised assay and all samples were subsequently above the LLOQ. All MEMO patients were, therefore, correctly identified as adherent when measured using the optimised index test.

For further validation from an independent cohort, 159 6-month RAMS samples were available. Following quality control, 138 samples remained. Out of 138 samples, only 7 were below the adherence limit (table 3) resulting in sensitivity of 95% from real-world self-reported patient samples.

## Discussion

Evidence consistently suggests that RA medication adherence is low in adults.^17^ Identifying non-adherent patients who can be targeted for supportive intervention is a clinical challenge. Often the prescriber is unable to determine if a patient is adherent and there is no gold-standard method developed to monitor adherence. Current National Institute for Health and Care Excellence guidelines on medicines adherence suggest assessing non-adherence by asking the patient if they have missed any doses of medicines recently.^18^ The use of indirect measures, such as self-reported questionnaires, has a number of challenges as patients may conceal their true behaviour to avoid being judged by their treating clinician.^19^

We present an HPLC-SRM-MS method developed and validated for the detection of MTX adherence. There are a number of strengths of the current assay: first, there is limited sample preparation required and that required is simple and straightforward compared with other assays; second, the assay has been shown to be sensitive for MTX detection and uses a technology that can be implemented in healthcare settings; third, the assay may be used in other disease in which low-dose MTX is prescribed such as psoriasis.^20^ A major advantage of HPLC-SRM-MS in therapeutic drug monitoring is its high sensitivity. As MTX is dosed weekly in standard rheumatology care, it was essential that the assay was sensitive enough to detect MTX several days after ingestion as, in routine clinical practice, patients will not always be seen at the same time following their MTX dosage. The development and validation of a PK model aided assay optimisation so that an assay sensitive enough to detect adherence in >80% of patients taking ≥5 mg of MTX weekly was developed. Both MTX and its major metabolite were studied, but MTX was found to be the superior analyte for the detection of MTX ingestion over the period of 1 week. The optimised assay demonstrated 100% sensitivity of all samples where direct observation of therapy was undertaken and 95% of samples from real-world self-reported patient samples. This is of vital importance so that clinicians can be confident in the result of an assay to detect adherence prior to discussing the assay results with the patient; the consequences of a false negative may be the erosion of trust in the patient–physician relationship. The assay was shown to be robust when analysing MTX naive plasma, demonstrating specificity.

There are a number of different methods which have been developed to measure MTX and its metabolites including assays that can detect MTX polyglutamate (MTXPG). However, in a recent study by Pasma *et al,* no correlation of measurement of MTXPG was found with a Medication Event Monitoring System that registers openings of the medication package; the findings did not, therefore, support the measurement of MTXPG as a biomarker of adherence.^21^ One possible explanation is the long t_1/2_ of MTXPG; the time when MTXPG levels become undetectable, can range from 2 to 32 weeks.^22^

While the sample size of the initial MEMO study was modest (n=20), the resultant PK profile of a two-compartment model has been suggested previously in several studies.^23–27^ Serum creatinine was not an informative covariate in this model, by contrast with the study by Godfrey *et al*.^28^ This may be due to the lack of creatinine variation in the population studied. While the MEMO study was observational and unable to control for concomitant therapies that may affect MTX PK, subsequent validation of the model, in a real-world study (RAMS), suggests that this is not clinically relevant as the developed MTX PK model performed well at predicting MTX concentrations.

Limitations of the current study include the fact that the assay can only detect whether the drug was taken and the correct dose within the preceding 6 days and does not reflect long-term adherence behaviours, and the assay would be unable to detect for example, patients who were regularly non-adherent but adherent in the few days preceding their appointments (so called white-coat compliance).^29^ Furthermore, we cannot exclude malabsorption as a factor in some patients to explain low serum levels rather than non-adherence. The study design limited the assessment of the negative predictive value of the test, and it is reassuring, however, that a patient with self-reported non-adherence was correctly identified as non-adherent. Detecting low drug levels and discussing this with patients does not necessarily mean that behaviours will be altered, although previous work in the field of hypertension has shown that screening for non-adherence to antihypertensive treatment using HPLC-SRM-MS analysis of urine/serum leads to subsequent improvement in measured adherence and blood pressure control.^10^ Specifically, Gupta *et al* measured antihypertensive drug levels in the urine and/or serum of hypertensive patients with feedback to patients of their results.^10^ Following feedback, the adherence ratio (the ratio of detected to prescribed antihypertensive medications) increased from 0.33 (IQR: 0–0.67) to 1 (IQR: 0.67–1) with an associated improved blood pressure control.

Further work to test the assay in a clinical environment is required to assess whether identification of MTX non-adherent patients will improve adherence and whether the intervention would be cost-effective. Low-dose MTX is the first-line drug for the treatment of RA and is used in other diseases such as psoriasis and psoriatic arthritis. Non-adherence to treatment may be a significant barrier to achieving full treatment response. If non-adherence is identified, support programmes could be considered as the use of a patient support programme to improve adherence to adalimumab has previously demonstrated greater adherence, improved persistence and reduced total healthcare costs.^30^

In conclusion, we have developed and validated an HPLC-SRM-MS assay to monitor MTX adherence. The assay has demonstrated a high sensitivity required for adherence detection to low weekly doses of MTX used in several chronic inflammatory conditions and the assay has been validated in independent real-world samples. The next vital work to implementation is a clinical trial to investigate whether measurement of MTX adherence using the assay can improve MTX adherence.
