# Supplementary material for: Development and validation of a methotrexate adherence assay
Source: Ann Rheum Dis. 2019 Jun 5;78(9):1192–7. doi: 10.1136/annrheumdis-2019-215446 (PMC6788879; doi:10.1136/annrheumdis-2019-215446)
Supplement: Supplementary data [file annrheumdis-2019-215446supp001.pdf]

Supplementary

**S1 Preparation of samples, chromatographic and mass spectrometry (MS) conditions**

Blank-free plasma and serum samples were stored at -80°C, thawed, spiked with MTX, 7-OH-MTX and the internal standard (MTX-$d_3$) and protein precipitated with methanol. Samples were centrifuged at 10,000g for 10 minutes, the supernatant removed and dried in a concentrator (Eppendorf concentrator plus) at room temperature and reconstituted in 50µl water prior to LC-MS/MS analysis. An autosampler auto-injector (Agilent 1200 series Autosampler with Thermostat, Cheadle, UK) was used to inject 5µl of sample. Chromatographic separation was performed using a Thermo Scientific Hypersil GOLD™ HPLC column maintained at 25°C on an Agilent 1200 series HPLC on-line to the MS (Agilent® 6460 triple quadrupole). The mobile phase consisted of acetonitrile (ACN) with 0.1% formic acid (B) as the organic component and water with 0.1% formic acid at the aqueous phase (A), the gradient elution timetable is shown in Table S1.

| **Time after injection (min)** | **Buffer A (%)** | **Buffer B (%)** |
|---|---|---|
| 0.00 | 92 | 8 |
| 1.00 | 92 | 8 |
| 4.00 | 50 | 50 |
| 5.50 | 50 | 50 |
| 5.60 | 5 | 95 |
| 8.00 | 5 | 95 |
| 8.21 | 92 | 8 |
| 11.00 | 92 | 8 |

**Table S1. Gradient elution timetable.**

The MS operated in positive ion mode and MTX/7-OH-MTX were detected using the following SRM transitions: 455.1 > 308.1 for MTX, 471.1 > 324.1 for 7-OH-MTX, and 458.1 > 311.1 for MTX-$d_3$ as described previously (1).

**S2 MTX Assay Optimisation and Re-evaluation of Adherence Measurement**

The HPLC-SRM-MS assay was optimised by discarding 7-OH-MTX measurement and increasing the MTX transition dwell time. The subsequent LLOQ of the optimised assay was 0.1 nM with precision (CV%) ≤3.5%, accuracy was within ±20% and signal:noise ratio > 10 in serum and plasma.

**S3 Recruitment flow chart for the MEMO study from RAMS**

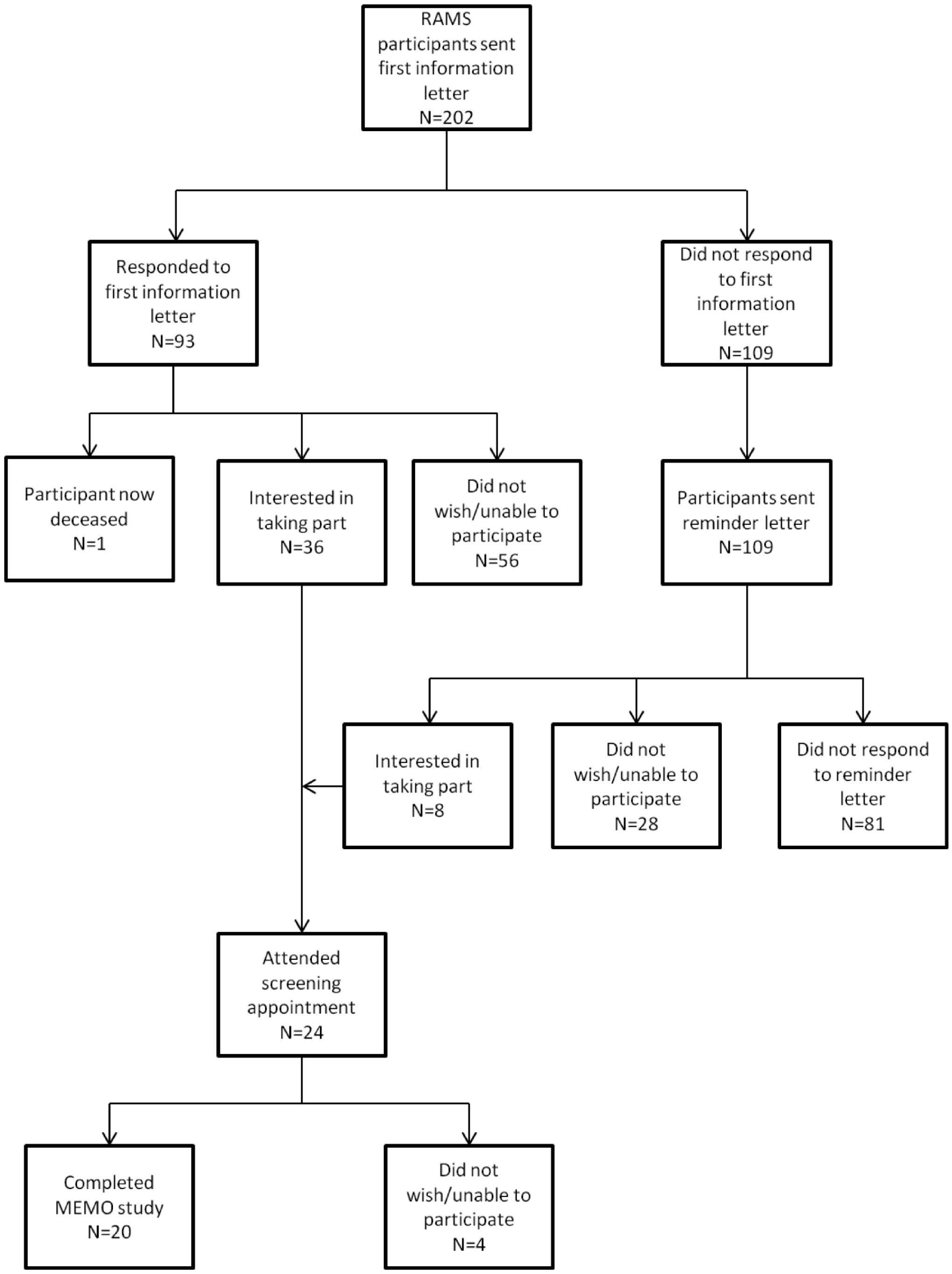

RAMS participants sent first information letter N=202
Responded to first information letter N=93
Did not respond to first information letter N=109
Participant now deceased N=1
Interested in taking part N=36
Did not wish/unable to participate N=56
Participants sent reminder letter N=109
Interested in taking part N=8
Did not wish/unable to participate N=28
Did not respond to reminder letter N=81
Attended screening appointment N=24
Completed MEMO study N=20
Did not wish/unable to participate N=4

**S4. Compartment models used to fit the plasma data for MTX and 7-OH-MTX.**

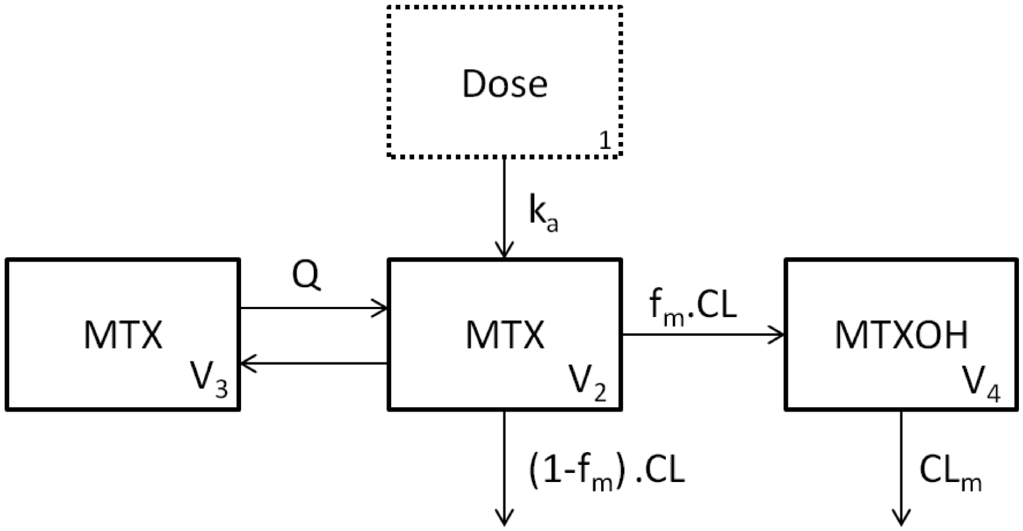


$K_a$ = absorption rate constant, CL = apparent MTX clearance, $CL_m$ = apparent 7-OH-MTX clearance, V = volume of distribution, $f_m$ = apparent fraction of MTX converted to 7-OH-MTX and Q = intercompartmental clearance. Compartment V4 was not structurally identifiable therefore apparent formation and clearance of 7-OH-MTX were estimated ($f_m$/V4 and $CL_m$/V4 respectively).

**S5. Population mean results from the pharmacokinetic modelling and the amount of variability within the model that is explained by each parameter.**

| **Parameter** | **Estimate** | **Relative Standard Error (%)** |
|---|---|---|
| *Population mean* | | |
| CL/F (l/h) | 6.75 | 7.0 |
| $V_2$/F(l) | 30.3 | 9.9 |
| Q/F (l/h) | 0.449 | 20.2 |
| $V_3$/F(l) | 65.8 | 46.0 |
| $k_a$ ($h^{-1}$) | 1.19 | 24.1 |
| $f_m/V_4$ ($l^{-1}$) | 0.00439 | 15.4 |
| $CL_m/V_4$ ($h^{-1}$) | 0.0563 | 8.2 |
| *Variability (CV%)* | | |
| CL/F | 20.2 | 49.0 |
| $V_2$/F | 24.2 | 63.2 |
| Q/F | 36.3 | 79.0 |
| $V_3$/F | 45.7 | 116.0 |
| $k_a$ | 42.3 | 107.0 |
| $f_m/V_4$ | 57.8 | 41.6 |
| $CL_m/V_4$ | 22.4 | 60.0 |

CL = clearance. F = bioavailability. Q = intercompartmental clearance. V = apparent volume of distribution. Ka = absorption rate coefficient, fm = apparent fraction of MTX converted to 7-OH-MTX, CLm = apparent clearance of 7-OH-MTX.

**Supplementary S6. Simulated data of 1,000 hypothetical individuals showing the proportion of subjects with predicted concentrations of MTX above the LLOQ following ingestion of 5mg (a), 7.5mg (b), 10mg (c), 12.5mg (d), 15mg (e), 17.5mg (f) and 20mg (g) MTX orally given LLOQ values as shown in the inserted box.**

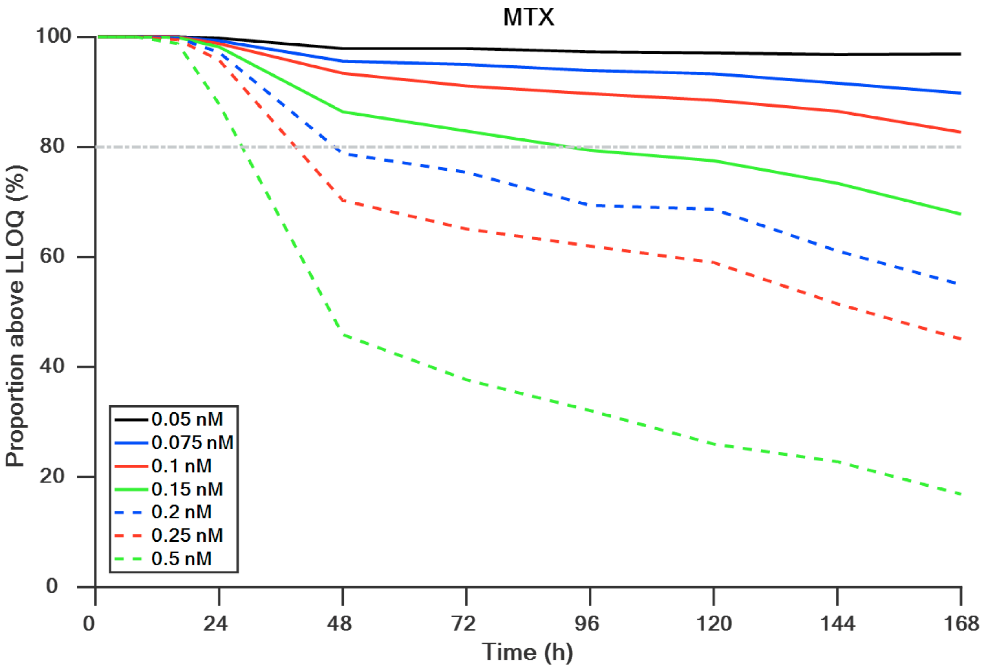


S6a)

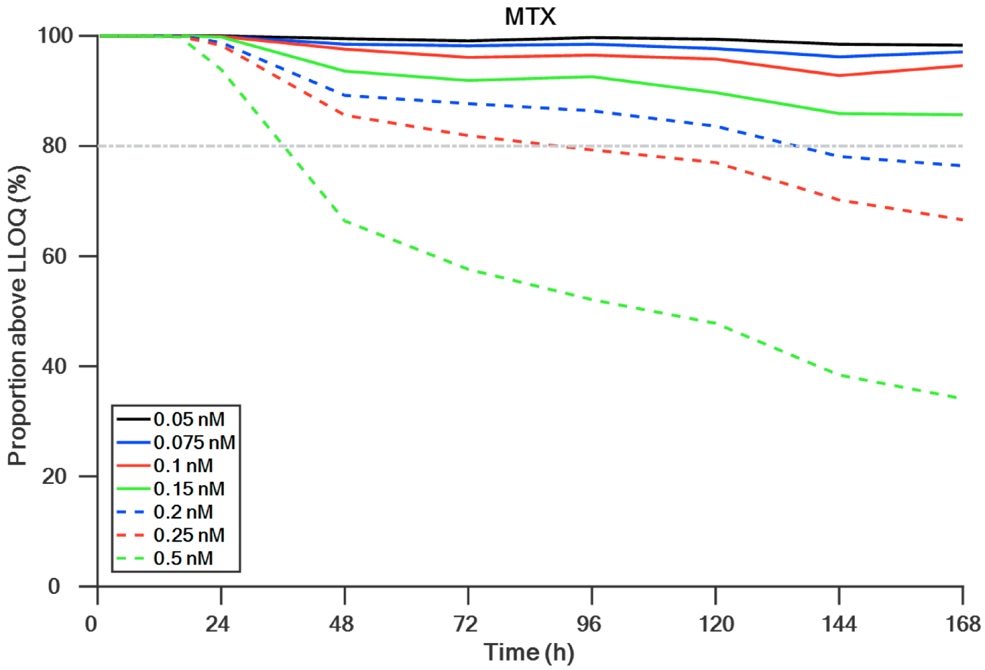
MTX
Proportion above LLOQ (%)
100
80
60
40
20
0
0
24
48
72
96
120
144
168
Time (h)
0.05 nM
0.075 nM
0.1 nM
0.15 nM
0.2 nM
0.25 nM
0.5 nM

S6b)

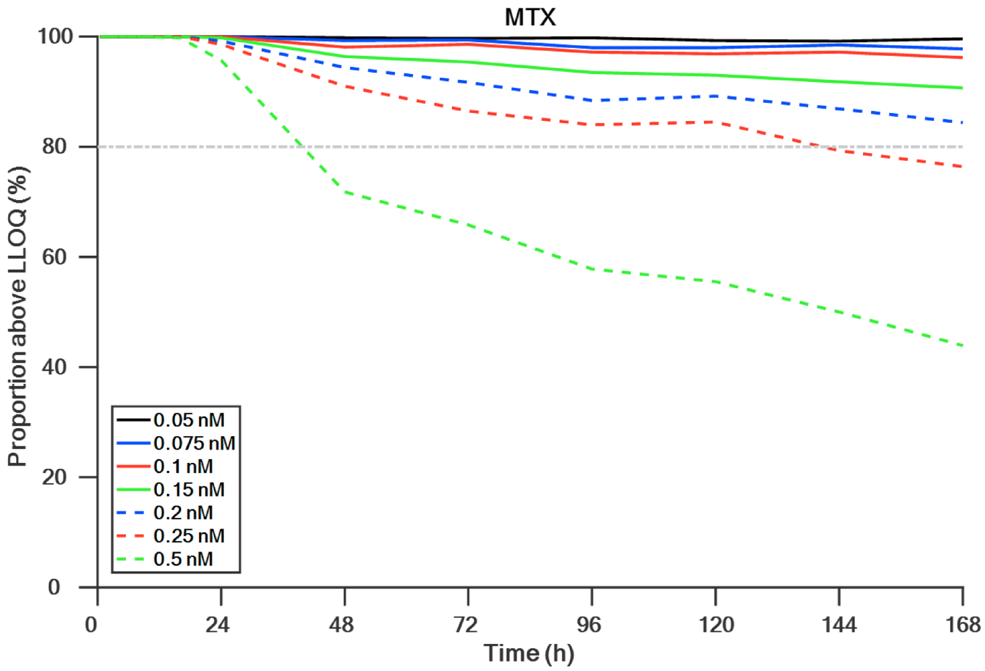
MTX
Proportion above LLOQ (%)
100
80
60
40
20
0
0
24
48
72
96
120
144
168
Time (h)
0.05 nM
0.075 nM
0.1 nM
0.15 nM
0.2 nM
0.25 nM
0.5 nM

S6c)

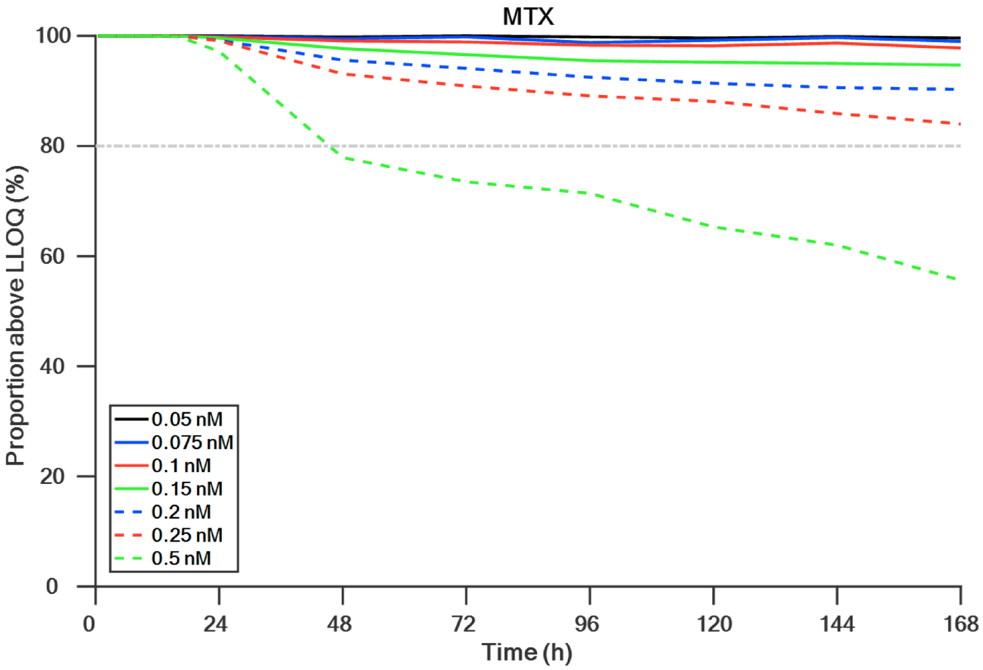
MTX
Proportion above LLOQ (%)
100
80
60
40
20
0
0.05 nM
0.075 nM
0.1 nM
0.15 nM
0.2 nM
0.25 nM
0.5 nM
0
24
48
72
96
120
144
168
Time (h)

S6d)

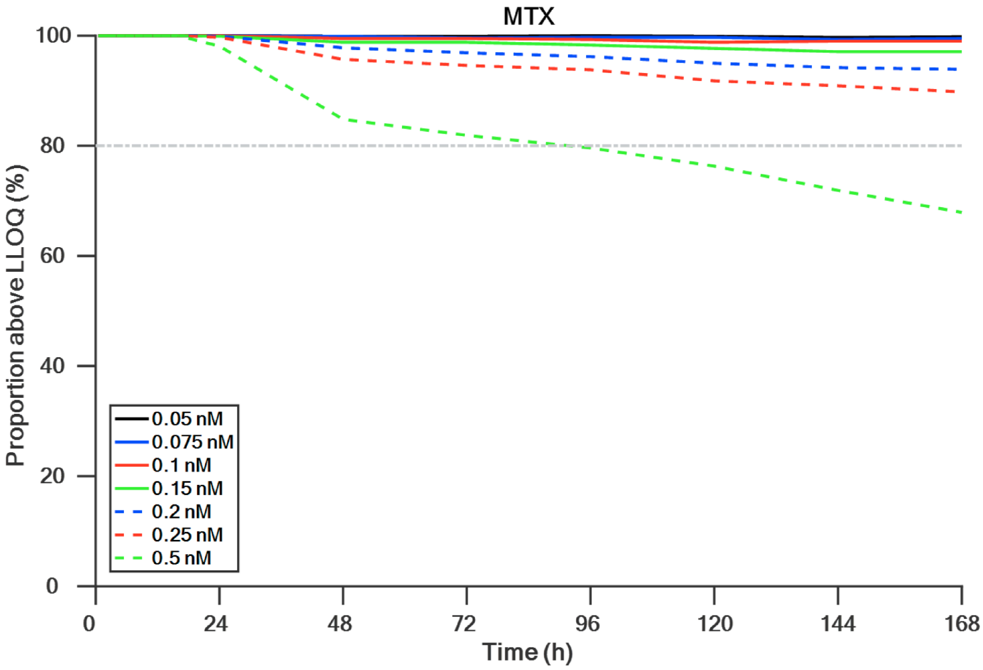
MTX
Proportion above LLOQ (%)
100
80
60
40
20
0
0.05 nM
0.075 nM
0.1 nM
0.15 nM
0.2 nM
0.25 nM
0.5 nM
0
24
48
72
96
120
144
168
Time (h)

S6e)

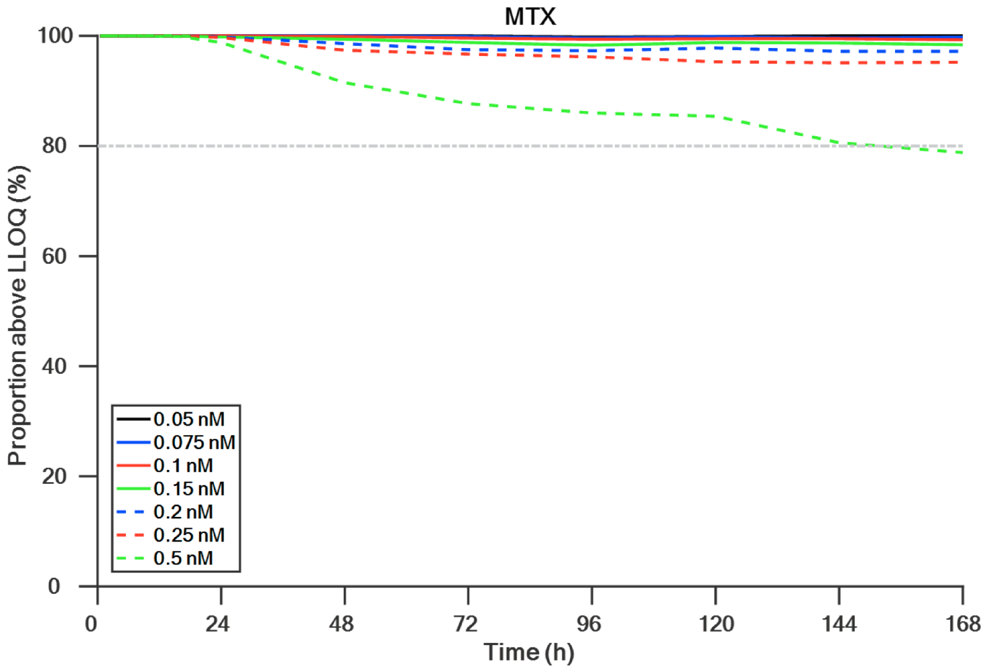


S6f)

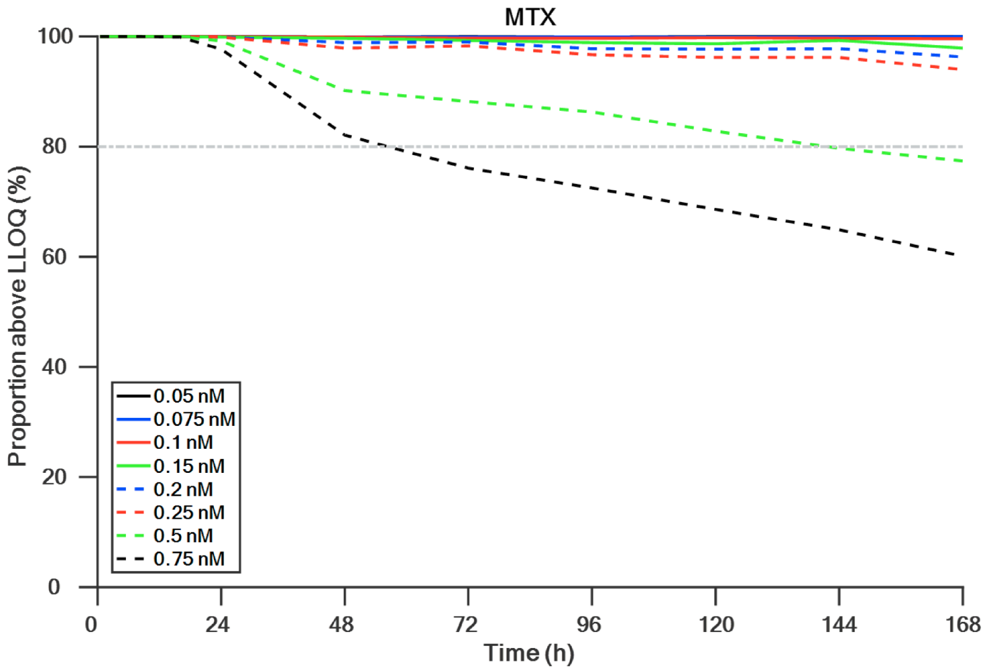


S6g)

**Supplementary References**

1. Bluett J, Riba-Garcia I, Hollywood K, Verstappen SMM, Barton A, Unwin RD**.** A HPLC-SRM-MS based method for the detection and quantification of methotrexate in urine at doses used in clinical practice for patients with rheumatological disease: a potential measure of adherence. Analyst. 2015;140(6):1981-7.
